# Differentiation of Types of Visual Agnosia Using EEG

**DOI:** 10.3390/vision2040044

**Published:** 2018-12-18

**Authors:** Sarah M. Haigh, Amanda K. Robinson, Pulkit Grover, Marlene Behrmann

**Affiliations:** 1 Department of Psychology, Carnegie Mellon University, Pittsburgh, PA 15213, USA; 2 Center for the Neural Basis of Cognition, Carnegie Mellon University, Pittsburgh, PA 15213, USA; 3 Department of Psychology and Center for Integrative Neuroscience, University of Nevada, Reno, NV 89557, USA; 4 ARC Centre of Excellence in Cognition and its Disorders, Department of Cognitive Science, Macquarie University, Sydney 2109, Australia; 5 School of Psychology, The University of Sydney, Sydney 2006, Australia; 6 Department of Electrical and Computer Engineering, Carnegie Mellon University, Pittsburgh, PA 15213, USA

**Keywords:** visual agnosia, EEG, decoding, SSVEP, neuropsychology

## Abstract

Visual recognition deficits are the hallmark symptom of visual agnosia, a neuropsychological disorder typically associated with damage to the visual system. Most research into visual agnosia focuses on characterizing the deficits through detailed behavioral testing, and structural and functional brain scans are used to determine the spatial extent of any cortical damage. Although the hierarchical nature of the visual system leads to clear predictions about the temporal dynamics of cortical deficits, there has been little research on the use of neuroimaging methods with high temporal resolution to characterize the temporal profile of agnosia deficits. Here, we employed high-density electroencephalography (EEG) to investigate alterations in the temporal dynamics of the visual system in two individuals with visual agnosia. In the context of a steady state visual evoked potential paradigm (SSVEP), individuals viewed pattern-reversing checkerboards of differing spatial frequency, and we assessed the responses of the visual system in the frequency and temporal domain. JW, a patient with early visual cortex damage, showed impaired SSVEP response relative to a control group and to the second patient (SM) who had right temporal lobe damage. JW also showed lower decoding accuracy for early visual responses (around 100 ms). SM, whose lesion is more anterior in the visual system, showed good decoding accuracy initially but low decoding after 500 ms. Overall, EEG and multivariate decoding methods can yield important insights into the temporal dynamics of visual responses in individuals with visual agnosia.

## 1. Introduction

Visual agnosia is a neuropsychological disorder characterized by severe difficulties in the recognition of common, everyday objects, and is not due to impairments in early sensory vision, memory or language function (for a review, see [1,2]). Visual agnosia (‘agnosia’ for short hereafter) is a highly heterogenous disorder that results from damage to a premorbidly normal region or regions of the cortical visual system, and is often referred to as ‘acquired’ visual agnosia. Agnosia can also result from brain damage that occurs in young children and is termed acquired ‘developmental’ agnosia (for review, see [3,4]) and can even be present in cases of ‘congenital’ agnosia in which behavior is impaired presumably from birth but without obvious neurological damage (for example, see [5]). The manifestation of deficits in acquired agnosia in adults (and perhaps in the other variants too) varies depending on the size and location of the lesion [6]. 

The classification of the behavioral profile of agnosia has traditionally been binary although more recently, there is growing recognition that the impairment may arise from any one of many transformations computed by the visual system. Standardly, the term ‘apperceptive agnosia’ refers to those individuals who have profound perceptual deficits resulting from a lesion to earlier parts of the visual cortex at which the elementary features of the stimulus are processed and a coarse structural description of the input is derived. The lesion typically associated with this form of agnosia results from comprehensive and widespread damage to the occipital cortex [7,8], often resulting from an anoxic episode or poisoning from mercury or lead [6]. Unsurprisingly, a person with apperceptive agnosia is markedly impaired on object recognition but also fails on a host of other basic tasks such as copying a target stimulus or matching a target with one of several choice objects. Apperceptive agnosia is sometimes differentiated from ‘visual form agnosia’ although there is some debate regarding this distinction [6,9].

At the opposite end of the agnosia continuum is ‘associative agnosia’ in which patients with damage to more rostral parts of the ventral cortex may be able to derive complex representations from the visual input but still cannot use the perceptual representation to access the stored knowledge of the object’s meaning and function. These individuals are able to draw from memory as well as copy and match target objects. More recently, a third category ‘integrative agnosia’ that likely falls intermediate between the binary extremes refers to an impairment to mid-level vision computations [10]. Individuals with this last form of agnosia may be able to copy and match a target object, but this is executed in a slow, piecemeal, and laborious fashion, perhaps feature-by-feature, and grouping disparate elements of the display into a coherent whole is abnormal [11].

Aside from the behavioral studies, most investigations conducted on individuals with visual agnosia focus on the localization of the underlying neural damage, and the consequences for the preservation or disruption of various visual behaviors. Either structural magnetic resonance imaging (MRI) scans, perhaps acquired at the hospital, or MRI scans, both structural and functional, obtained in research settings have served as the evidence for the site and size of the cortical lesion (for example, [12,13,14,15]). Somewhat surprisingly, even though we understand that there is a time course associated with processing signals in the visual system, to date, there have been remarkably few studies that have investigated the temporal dynamics of perturbed neural processing in visual agnosia. 

### 1.1. Visuoperceptual Deficits and Electrophysiology

Here, we argue that there is much to be learned from employing electrophysiological approaches to examine the temporal dynamics of visual processing, including the time points of perturbation in the waveform relative to the controls, and the downstream consequences of altered temporal dynamics for perception. Findings from such studies can further inform basic science questions by offering new insights into how early visual processing contributes to complex object processing and can provide insight into translational questions by elucidating the electrophysiological disturbances following brain damage in adulthood. 

The few studies that have employed high temporal resolution electroencephalography (EEG) have been informative. For example, several evoked-response potential (ERP) investigations have focused on face processing in individuals with face recognition deficits (prosopagnosia) and have examined the N170 (for a summary, see [16]) or the N250 and P600f components [17,18]. Key findings from these studies reveal that the presence or absence of the N170 waveform in individuals with acquired prosopagnosia corresponds to the locus of the lesion. That is, if the damage is to earlier regions of the visual cortex, then the N170 may be absent [19]. If, however, the damage is to the fusiform face area (FFA), then, unlike in the controls, the N170 does not evince a face inversion effect [20] and is less selective in that the signals do not differentiate between face versus non-face object stimuli. In one illustrative case, patient PS, with a lesion to the right inferior occipital gyrus, an early face-sensitive component was observed at approximately 170 ms post-stimulus over the right occipitotemporal region [21]. In the left hemisphere in which PS has a lesion in the middle fusiform gyrus, no N170 component was obtained, and these findings were replicated using magnetoencephalography. Related studies in individuals with prosopagnosia have focused on the P300 ERP to rare faces and have shown intact novelty detection [22], even when earlier visual ERPs such as the P100 are slower than those elicited from the healthy controls [23]. We also note that, just as in acquired prosopagnosia, there have been studies using ERP in individuals with congenital prosopagnosia and, again, these studies have focused primarily on the N170 component [20,24,25].

Last, there have been a few studies that have explored ERPs using stimuli other than faces. For example, one study examined ERPs to objects, faces, and Japanese and Chinese symbols in a single male with acquired associative agnosia believed to be related to his multiple sclerosis [26]. In this case, both the P190 and N170 to objects and faces were reduced compared to the controls, and P190 in response to symbols was smaller compared to control individuals. Another study reported ERPs to faces, watches and flowers in a patient with developmental visual agnosia [27]. Even though non-face stimuli are used in these studies, the focus of these papers was primarily on differences in the N170 in response to face stimuli in the single case/s compared to the controls with most emphasis on the N170 waveform for faces. 

As evident from this brief overview, the investigations conducted to date focus almost exclusively on event-related potentials (ERPs), which are neural components of the waveform reflecting an aggregate of activity over a period of time, and almost exclusively on the N170 component. Furthermore, the data are typically analyzed using univariate analyses to investigate visual responses (i.e., signal amplitude) by comparing the electrophysiological signal at particular components (like the N170) for upright versus inverted faces or for faces versus objects. This approach potentially obscures more subtle aspects of the electrophysiological signature that might be more apparent with multivariate analyses that assess patterns of activity. The use of multivariate statistical techniques as applied to EEG data has been advocated since 1978 [28] and, notwithstanding the fact that this may have more sensitivity than standard univariate ERP analyses, it has been used only infrequently (for example, [29]). Additionally, more robust approaches have been advocated recently and a particular advantage of this approach for neuropsychological investigations is that, with enough data, characterization of neural patterns within single subjects is possible. In the current study, we utilize the steady-state visual evoked potential (SSVEP) approach, which generates frequency information as well as temporal information in the EEG signal, in conjunction with multivariate analyses both to compare the patient electrophysiological profiles with that of the controls but also to differentiate between the two patients themselves.

### 1.2. The Current Study

The current study investigated the temporal dynamics of visual processing in two individuals with acquired visual agnosia following damage to different parts of the visual system, and compared them to each other as well as to a group of control participants. Patient JW who has apperceptive agnosia sustained damage bilaterally to primary visual cortex (V1) whereas SM sustained damage to the lateral-inferior temporal lobe (further details below) and his profile is more similar to integrative or even associative agnosia. 

The goal of this paper is to exploit the high temporal resolution afforded by the EEG methodology to examine at what time points the visual signal was normal in visual agnosia, when the signal was degraded due to the acquired damage and what abnormalities characterized the waveforms. Furthermore, to permit fine-grained detection of differences between the patients and controls, the entire time course of the EEG signal across all electrodes was examined and decoded as a means of documenting the response profile of the patients compared with control individuals. 

**Figure 1:**
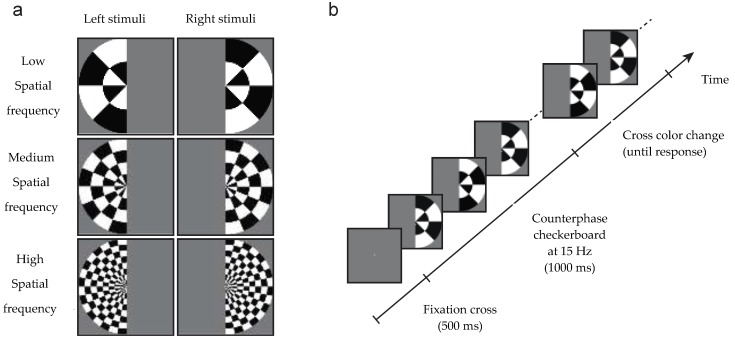
Stimuli and design of the experiment. (**a**) Stimuli were low, medium or high spatial frequency checkerboards presented in the left or right visual field. (**b**) A central grey fixation cross was presented for 500 ms, then the checkerboard stimulus counter-phased at 15 Hz to generate a steady state visual evoked potential paradigm (SSVEP). After 1000 ms, the cross became red or green, at which time participants discriminated its color and responded via a key-press.

This study takes advantage of the findings from Robinson et al. [30] in which we used decoding analyses in typical individuals to measure EEG responses to checkerboard stimuli (see Figure 1). In that study, we used checkerboard displays that differed in spatial frequency (low, medium and high) that could appear in either the left or the right visual field. Highest decoding accuracy (frequency × field; six-way) was observed around 100 ms, and this peak decoding accuracy was even evident on an individual-by-individual basis. To uncover the neural correlate of the signal at 100 ms, we compared the stimulus representations to a computational model of V1, and observed a significant correlation, supporting the claim that the EEG signal we decoded was likely the output of area V1. The decoding accuracy also remained above chance for the remainder of the time that the flickering stimulus was presented although the accuracy was lower, suggesting that as long as the stimulus was presented, early visual areas were processing the stimulus. This experiment and method of analysis offers us the opportunity to analyze the reliability of the EEG signal and benchmark the temporal profile, relative to a control sample, in two agnosic patients with lesions to different sites within the cortical visual system. The use of a checkerboard pattern ensures a strong EEG signal that can be easily decoded over time. In addition, checkerboard stimuli are processed initially in V1 but continue to be processed throughout the visual system, allowing for comparisons between early visual cortex and later more complex visual processing areas. Other stimuli such as objects rely on higher-level processes in the visual system and so may not be as sensitive to determining early impairments within V1.

**Figure 2:**
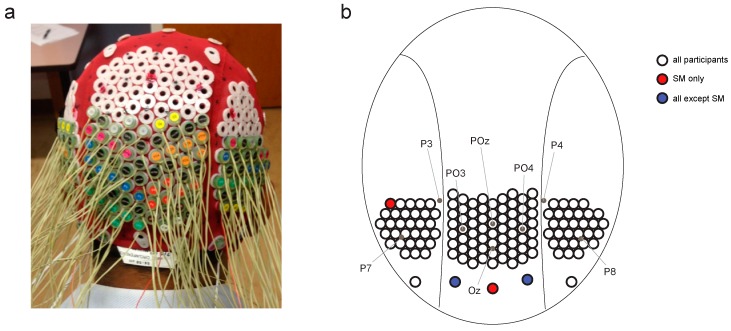
Head cap configuration. (**a**) Modified electroencephalography (EEG) head cap with high-density electrode configuration. (**b**) Map of electrode configuration on the back of the head, showing approximate locations of electrodes from the 10–20 standard configuration.

In the current study, we adopted the same stimulus protocol and similar electrode configuration as reported by Robinson et al. ([30]; see Figure 1 and Figure 2). The hypothesis is that the decoding accuracy of the EEG signal will differ between the two patients and that the patients’ profiles will also differ from the controls. The more specific prediction is that JW’s EEG responses should result in poor decoding early in the time course, consistent with damage to early visual areas, whereas SM’s responses should initially be reliably decoded, but that the decoding accuracy should be at chance levels later in time, consistent with his intact V1 functions but damage to later visual areas. Support for these predictions will provide justification for the increased use of EEG systems in individuals with neuropsychological disorders and will further support the basic science use of EEG as a means of carefully documenting the microgenesis of neural signals across cortex.

## 2. Materials and Methods

### 2.1. Participants

Two male individuals with acquired visual agnosia, JW and SM, were recruited for this study and each is described in turn. Both patients exhibit deficits in being able to visually recognize objects [31,32,33,34].

**Figure 3:**
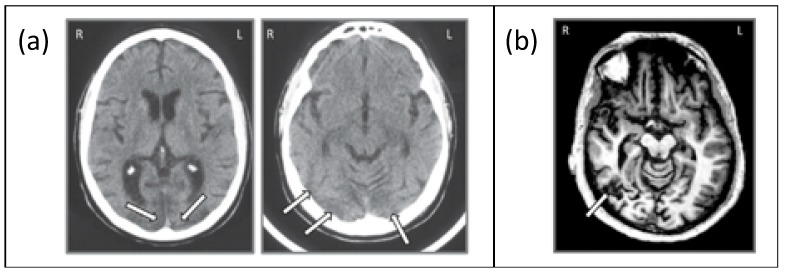
(**a**) Axial slice from the CT scan of JW showing bilateral posterior damage to parts of V1 and V2 and (**b**) axial slice from the MRI scan of SM showing lateralized damage to the right inferior temporal lobe. Adapted from Freud et al. [8], with permission from Oxford University Press, 2018.

JW (59 years old) acquired agnosia at age 35 due to anoxic encephalopathy associated with a cardiac valve abnormality. Based on a computed tomography (CT) scan, JW was diagnosed with generalized atrophy and ischemic infarction in both occipital cortices, extending slightly into the right parietal lobe and the extensive ischemic wrap around the occipital pole revealed widespread V2 and V1 injury ([35,36,37,38]; see Figure 3a). This diagnosis was provided independently by two neuroradiologists, neither of whom reported any damage affecting higher-level cortices. Unfortunately, we have not been able to obtain an MRI scan on JW as he has a prosthetic metal sternum, inserted during cardiac surgery. JW has recovered functioning in non-visual tasks such as language and movement and shows no memory loss. He has corrected retinal vision but has an incomplete scotoma in his upper left visual field. JW has some impairment in low-level visual tasks such as contrast sensitivity as well as difficulties in grouping, segregating figures from the background, segmenting and discriminating size and shape, as well as difficulties in contour integration and perceiving symmetry [31,35]. He also shows impaired face recognition and word recognition (perhaps unsurprising given the difficulties in signal processing in early visual areas). However, he shows intact object memory and imagery, color vision, visual-motor control [33], and spatial attention [37].

SM (42 years old) acquired agnosia at age 18 as a result of a motor vehicle accident that resulted in a lesion of the right inferior temporal lobe in the vicinity of the lateral occipital complex ([8,39], see Figure 3b). Interestingly, a functional MRI study revealed that not only was the BOLD signal in the lateral occipital complex (LOC) region in the right hemisphere abnormal but a similar abnormality was evident for the LOC region in the left hemisphere, suggesting that the impaired right side may suppress or inhibit the homologous region in the structurally preserved hemisphere [13]. SM recovered almost completely except for the visual agnosia and some weakness in his right arm and leg, which were injured in the accident. Unlike JW, SM has normal performance on low-level visual tasks such as judging line length, orientation, size, and gap position, has normal color vision, and can copy drawings of objects and scenes. He has 20/20 vision and no ophthalmological abnormalities. He can also read accurately but does so more slowly than healthy individuals. Object naming through haptic or auditory modalities is normal. However, face recognition, object categorization, and discriminating between overlapping objects are impaired [37,38]. 

Therefore, while both individuals have visual agnosia, JW’s agnosia resulted from damage more posteriorly and bilaterally in the visual system while SM’s damage is to more anterior structures and in the right hemisphere. These contrasting behavioral and neurological profiles provide the opportunity to investigate how the time course of the visual signal differs depending on the location of the damage.

The control data were taken from Robinson et al. [30]. Twenty participants (18 male) from the Pittsburgh area were recruited. Four participants were excluded due to excess eye movements or poor data quality, leaving *N* = 16. 

All participants, including the patients, were compensated US $50 for their time. The study was conducted at Carnegie Mellon University and was approved by the University’s Institutional Review Board (00000465). All participants gave their informed consent and the study was conducted in accordance with the Declaration of Helsinki.

### 2.2. Design and Stimuli

Stimuli were identical to those used by Robinson et al. [30]. Stimuli consisted of low, medium or high spatial frequency checkerboards with a radius of 28 degrees of arc that appeared in either the left or the right visual field (see Figure 1). The spatial frequencies radially were 0.036, 0.071 and 0.143 cycles per degree (equivalent to 1, 2 and 4 wavelengths of alternating black/white tiles), and 0.011, 0.022 and 0.044 cycles per angular degree respectively (equivalent to 2, 4 and 8 wavelengths in a semicircle). To generate a SSVEP, the contrast of the checkerboards reversed at a frequency of 15 Hz for 1 s. Spatial frequency and hemifield were sampled with equal probability in each block of trials. Following the 1 s checkerboard, the central fixation cross became red or green. Participants performed a simple orthogonal task: to discriminate the color of the fixation cross using key presses (and both JW and SM have normal color discrimination). No feedback was given regarding accuracy.

The Psychophysics Toolbox in Matlab was used to present the visual stimuli on a 24-inch LCD monitor with 60 Hz refresh rate. There were 100 repeats of each of the 6 stimulus types split into ten blocks of trials, resulting in 600 trials for the whole experiment. Participants could take a break between blocks. Due to a script crash during one block, only 552 trials were collected from SM rather than the 600 from JW and control subjects. After rejection of bad trials (see Section 2.4), however, trial numbers were roughly comparable between the controls and patients. 

### 2.3. EEG Recording

Continuous EEG data were recorded using a BioSemi Active Two system (BioSemi, Amsterdam, Netherlands), digitized at a 512 Hz sample rate with 24-bit A/D conversion. The 128 electrodes were approximately 14 mm apart and were arranged using the same nylon head cap and custom high-density configuration over the back of the head as Robinson et al. [30] (Figure 2). Note that the placement of two of SM’s peripheral electrodes varied subtly compared with JW and the controls, but this small difference was unlikely to drive differences in the temporal dynamics of visual processing. All scalp electrodes were recorded relative to the standard BioSemi CMS and DRL electrodes, and were re-referenced offline to the average of the 128 electrodes. Eye movements were monitored using bipolar horizontal EOG electrodes placed at the outer canthi of each eye and bipolar vertical EOG electrodes placed above and below the left eye. Trials were excluded if the difference between the horizontal eye electrodes exceeded 100 μV between −100 and 300 ms from stimulus onset.

### 2.4. EEG Preprocessing

EEG data were analyzed using EEGlab [40] and preprocessed using the PREP pipeline [41]. The preprocessing steps were as follows: data were high pass filtered at 1 Hz, line noise was filtered and were average referenced. No electrodes were interpolated. The data were then subjected to low-pass (0.1 Hz) and high-pass (100 Hz) zero-phase filters. To ensure that the results were not due to non-brain artefacts, we conducted the same analyses using more stringent filters (10–20 Hz). The results were similar and are included in Supplementary Material Figure S3. Epochs were constructed relative to the onset of the checkerboards and baseline corrected for 200 ms prior to onset. The epochs were then subsampled to 256 Hz using the decimate function in Matlab. For each participant, trials were excluded if they had any horizontal eye movements from −100 to 300 ms from image onset, or contained more than 10 channels exceeding 150 uV over the course of the trial. This step was to eliminate the noisiest trials; decoding analyses are particularly good at dealing with bad trials and thus, a stringent trial rejection step was unnecessary [42,43]. See Supplementary Material Figure S1 for mean event-related potentials per stimuli visual field for each of the patients and the controls. 

### 2.5. Frequency Domain Decoding

A support vector machine (SVM) classifier was used to decode the neural activity evoked by the six different stimuli from the SSVEP response. Decoding was performed for all pairs of stimulus types (frequency X field), for example, right visual field (RVF) low spatial frequency vs left visual field (LVF) low spatial frequency and RVF low spatial frequency vs. LVF med spatial frequency. This resulted in 15 pairwise classification tests with chance performance of 50%. Note that in Robinson et al [30], we assessed 6-way classification accuracy (chance = 16.67%), but here we analyze pairwise decoding in order to obtain accuracy distributions across the 15 pairs.

After the elimination of bad trials, trial numbers were equated across stimulus classes per participant by taking the highest even number available for all classes (controls: min = 82, max = 100 trials per class; both patients: 90 trials per class). Two trials per stimulus class were averaged before classification (chosen randomly across all trials for that stimulus) to reduce non-stimulus related EEG noise. This pseudotrial technique has been shown to improve classification performance [42] and also increases the signal-to-noise ratio for SSVEP signals. For frequency domain analysis, a discrete Fourier transform was applied to the averaged pseudotrials from 0–1000 ms using the FFT function in Matlab. The amplitude at 15 Hz was selected for each electrode and pseudotrial, resulting in a matrix with dimensions 128 × *P*, representing the 15 Hz amplitude for each electrode over *P* pseudotrials. These data were *z*-scored across trials, and principal components analysis (PCA) was performed to reduce data dimensionality. These data were then fed to the classifier. For each pair of stimuli, an SVM classifier was used with 9-fold cross-validation. At each fold of cross-validation, PCA was performed on the training data and weights were applied to the test data [42]. PCA components accounting for 99% of the variance in the training data were used as features for classification. 

### 2.6. Time Course Decoding

Similar to the frequency domain decoding, a support vector machine (SVM) classifier was used to decode the neural activity evoked by the six different stimuli. These analyses were performed in the temporal domain, at each time point from stimulus onset. The same preprocessing and decoding parameters were used with respect to pseudotrial construction, z-scoring and PCA feature selection. Analyses were conducted for each participant and time point separately.

### 2.7. Statistical Analysis

Three critical statistical tests were implemented per decoding analysis to assess (1) the significance of each patient’s classification compared to chance, (2) the significance of each patient’s classification compared to the control group, and (3) the comparison of the two patients to each other.

First, the obtained individual patient classification accuracy was compared to a null permutation distribution from their own data. Using each patient’s pseudotrial data, classification was performed 1000 times with shuffled class labels, producing a null decoding distribution with which to compare the decoding accuracy. The same randomization seed was used for each patient. This was done for each of the pairwise classification tasks, that is for 15 stimulus pairs, and averaged across pairs. For each time point or test, *p*-values were calculated as *p* = (*n* + 1)/(1000 + 1), where *n* was the number of permutations (out of 1000) that were more extreme than the obtained “real” decoding accuracy. For frequency domain decoding, obtained classification accuracy was considered to be significant if it exceeded all values in the null distribution (*p* < 0.001). For time course classification, the criteria for significance were more stringent; decoding was considered significant only if three subsequent time points exceeded all values in the null distribution, *p* < 0.001 (the assumption is that it is unlikely for a single time point to be significant if none of its neighbors is significant).

Second, the obtained classification accuracy per patient was compared to the control group. Z-scores were obtained for each of the agnosia patients compared with the control group. Absolute *z*-scores of more than 2 standard deviations from the control group are discussed. Again, for the time course analysis, patients are considered to differ if at least three subsequent time points are more extreme than the control group. Control group significance was assessed using one-sample *t*-tests comparing the group mean to chance performance (50%) at each time point. *p*-values were corrected using the Benjamini–Hochberg procedure for controlling the false discovery rate, for the 307 EEG time points that were assessed, and considered significant if *p* < 0.001.

Finally, the obtained individual classification accuracies were compared for the two patients. A null difference distribution was obtained by taking the difference between each value in JW’s null distribution and SM’s null distribution (for each time point and frequency domain decoding). This yielded a distribution by which JW and SM would be expected to vary due to chance. The obtained difference in decoding accuracy between JW and SM was then compared to this distribution, for the frequency domain decoding as well as time course decoding. Again, *p*-values were calculated as *p* = (*n* + 1)/(1000 + 1), and classification accuracy was considered to be significant for values *p* < 0.001.

## 3. Results

In this study, EEG was recorded from JW and SM while they viewed achromatic checkerboards that alternated in their contrast at 15 Hz, and the electrophysiological signals were compared to a group of individuals with no visual impairment as well as to each other. The checkerboards were presented in the left or right visual field and were low, medium or high spatial frequency, yielding six conditions of interest. Two main analyses were performed to compare the patient and control data: (1) decoding stimulus representations in the time-frequency domain, and (2) decoding stimulus representations across the time-course of a trial. Finally, the data from the two patients were compared to each other.

### 3.1. Frequency Domain Decoding

First, to test the six-way discrimination of the signals, we decoded stimulus responses in time-frequency space, capitalizing on the stimuli evoking steady state visual evoked potentials (SSVEP; [44]. A fast Fourier transform was applied to each averaged pseudotrial 0–1000 ms from stimulus onset, and the amplitude at 15 Hz (the stimulus flicker frequency) for each electrode was fed to the classifier. This analysis established whether the spatial SSVEP information captured by EEG can reliably distinguish between the stimuli.

**Figure 4:**
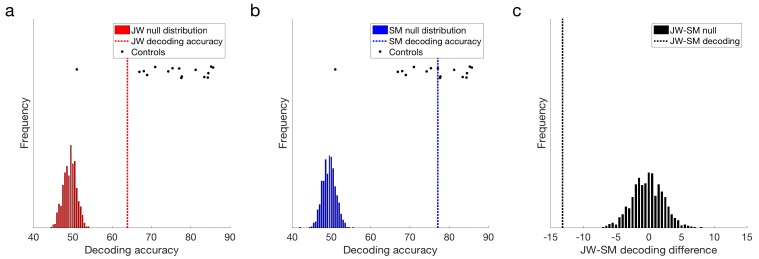
Frequency domain decoding results. (**a**) JW and (**b**) SM compared with their null distributions and individuals in the control group. Dotted lines designate the obtained classification accuracy for that patient. (**c**) Difference in decoding accuracy between JW and SM compared with the difference in the null distributions. JW’s frequency domain decoding was significantly lower than SM’s, *p* = 0.001. Note the single control outlier in the control distribution.

Both JW and SM showed above chance decoding at 15 Hz compared to their null distributions, *p* = 0.001 (Figure 4a,b). This result demonstrates that the flickering checkerboard stimuli were being encoded by the visual system of both patients. Frequency decoding for JW was lower than that of most of the controls: z-scores indicated that JW was 1.30 standard deviations below the control mean, whereas SM’s accuracy was still within the control range, *z* = 0.14. It should be noted, however, that there was one control that did not achieve significant decoding in the frequency domain (*M* = 50.62%), likely due to low SNR. Inclusion of this participant in the control distribution increased the group variance markedly. Excluding this participant, it was clear that JW’s decoding was significantly below the control group, *z* = −2.06, *p* = 0.040. SM was still not significantly different from the controls, *z* = −0.054, *p* = 0.957. Examination of decoding accuracy for stimuli pairs within a visual field (e.g., LVF low vs. LVF medium spatial frequency) and for the same spatial frequency across visual fields (e.g., LVF low vs. RVF low) revealed that mean decoding for both patients was not entirely driven by differences across visual field, and that accuracy in general was lower for JW than the controls (see Supplementary Material Figure S2). Together, these results suggest that JW had impaired SSVEP signals relative to the controls. 

Additional evidence for an impairment in JW’s visual processing came from comparing the signals of the two patients directly. As seen in Figure 4c, JW’s decoding was significantly below that of SM, *p* < 0.001. Overall, these results implicate JW’s early visual system impairments in the reduced stimulus-specific neural responses in the frequency domain. SM, in contrast, did not appear to have obvious processing deficits in the frequency domain. 

### 3.2. Time Course Decoding

Second, we examined the time course of decoding the stimuli. At each time point across the course of a trial, a linear support vector machine (SVM) classifier was used to decode the neural activity evoked by the different stimuli. Pairwise classification was performed for all pairs of stimuli (low, medium and high spatial frequency stimuli in left and right visual fields; 15 pairs). Chance decoding was at 50%.

**Figure 5:**
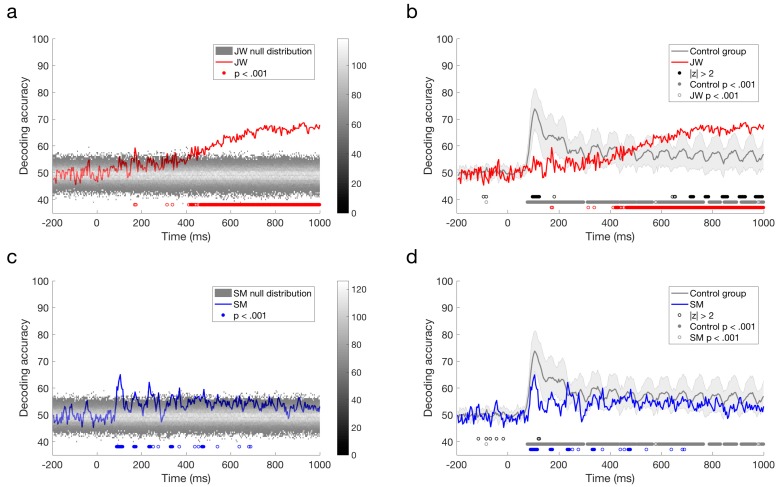
Time course classification results for visual agnosia patients. (**a**) JW’s decoding performance compared to shuffled label permutations. (**b**) JW’s decoding performance compared to the control group. (**c**) SM’s decoding performance compared to shuffled label permutations. (**d**) SM’s decoding performance compared to the control group. Error bars designate one standard deviation of the control group. Filled circles below plots a and c indicate significant decoding above chance, and open circles indicate those points that were not sustained for at least three successive time points. For plots b and d, filled black circles indicate significant decoding that was two standard deviations above or below the control group (**b**,**d**). Open black circles indicate those points that were not sustained for at least three successive time points. Grey circles indicate decoding that is above chance for the control group. Red (**b**) circles indicate decoding that is above chance for JW, and blue (**d**) circles indicate decoding that is above chance for SM.

The responses reported by Robinson et al. [30] with typical controls revealed peak decoding around 100 ms, corresponding with the P1 ERP component, which is likely to originate from the early visual cortex. As evident from a quick glance at Figure 5c,d, the profile elicited for JW bears little resemblance to that of the controls whereas SM’s profile is qualitatively similar to that of the controls. As can be seen in Figure 5a, the stimuli did not elicit a 100 ms peak for JW, although there was a suggestion of decoding at approximately 170 ms, and prolonged reliable decoding after ~400 ms. JW’s decoding appeared to be much lower than the controls in early time period, but higher than the controls at later time points. In contrast, SM showed reliable decoding at 90–116 ms post stimulus-onset, and there was no reliable decoding after 480 ms (Figure 5b,d). Although lower in accuracy overall, SM’s decoding was not reliably different from the control group. 

**Figure 6:**
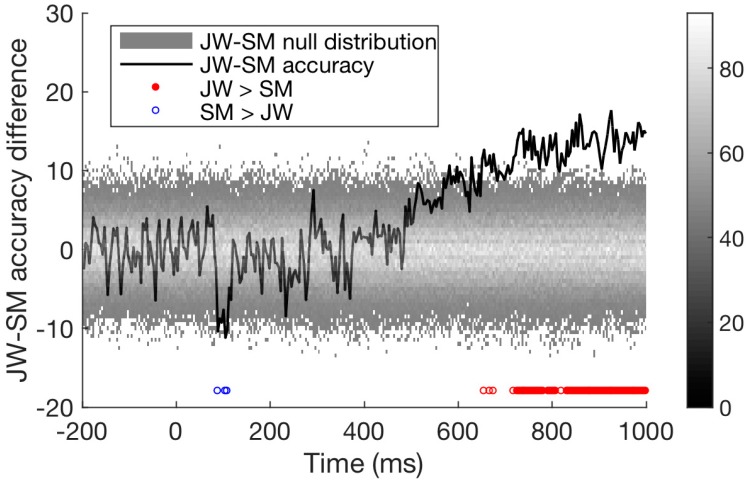
Difference in time course classification results for the two visual agnosia patients (JW minus SM). Accuracy for the two agnosia patients differed over time; around 100 ms, SM outperformed JW, but from 500 ms onwards JW had much higher decoding accuracy than SM, *p* < 0.01.

**Figure 7:**
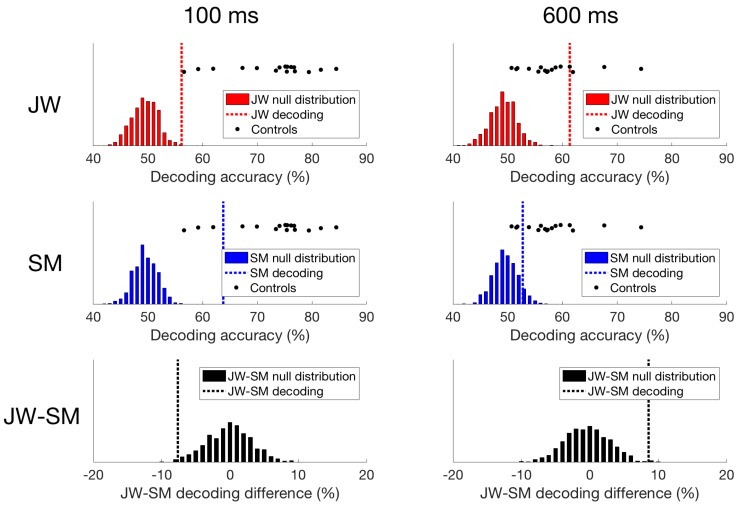
Time domain decoding results. For JW (top row), decoding at 100 ms was significant, but much lower than all of the controls. However, at 600 ms, JW’s decoding accuracy exceeded most of the controls highlighting a delay in visual processing. For SM (middle row), decoding at 100 ms was significant, and while decoding accuracy was still lower than most of the controls, his responses still fell within the control distribution. At 600 ms, SM’s decoding accuracy was not significant, and fell within the distribution of decoding from the controls. When comparing JW to SM (bottom row), JW’s decoding was poorer at 100 ms than SM’s, and the reverse is true at 600 ms.

Finally, we compared the time course of decoding for the two patients directly. As seen in Figure 6, decoding of JW’s data was below that of SM at 100 ms, at the time of the first peak. This is additional evidence of JW’s early visual processing impairment. SM’s decoding, however, was significantly lower than that of JW for later timepoints >600 ms. Together, these results echo the frequency domain results by showing that JW’s early visual system impairments reduced stimulus-specific neural responses. Later, higher level processing for SM appeared to be lower than JW but was within the range of healthy individuals. A summary of the time course decoding results can be seen in Figure 7.

To ensure that the patterns of results were not driven by systematic artifacts in the data, we repeated the analysis using data that was filtered using a more stringent strategy. Before epoching, data were filtering using a 10 Hz highpass and 20 Hz lowpass to remove any low-frequency artefacts such as eye movements, and higher frequency muscular artefacts. As can be seen in the Supplementary Material Figure S3, the new analyses revealed that JW had poorer decoding in the frequency domain than the controls and SM, and JW had poorer decoding early in the time course (100–200 ms) relative to the controls and SM. Thus, this stringent filtering analysis provides additional evidence for JW’s early visual processing impairment.

## 4. Discussion

We examined the EEG signal in two individuals with visual agnosia to characterize the electrophysiological response profile in agnosia and to ascertain whether the reliability of the signal might reveal the rough locus of the lesion site. We chose to focus on agnosia as the behavioral profiles of the two patients, JW and SM, have been widely documented offering a detailed understanding of preserved and impaired functioning. In addition, agnosia can be the result of damage to any of a number of region/s of the visual system. Understanding the time course of the visual signal may elucidate how some visual functioning remains intact despite damage to early visual cortical areas. We used the same protocol as described by Robinson et al. [30] allowing for a direct comparison of the two agnosia patients with healthy individuals as well as a comparison between the two individuals. 

### 4.1. Frequency Domain and Temporal Domain Decoding

Using decoding methods, we found that, in the frequency domain, both JW and SM produced steady-state visual signals that could be reliably decoded at 15 Hz, consistent with the oscillation rate of the checkerboard stimulus pattern. However, the accuracy of the frequency decoding was significantly poorer in JW compared to SM and to the controls. This demonstrates that, while the visual signal is clearly being encoded in both patients, the electrophysiological profile was not as robust in JW, suggesting that his impairments to the early visual cortex influenced the frequency domain responses. Indeed, pattern reversal SSVEP responses have been localized to the early visual cortex [45] and fast frequencies such as 15 Hz seem to bias processing to early visual stages relative to slower frequencies [46]. Also, as mentioned previously, this particular paradigm targets early visual areas and, in particular, V1, as evident from the significant high correlation of the stimulus dissimilarity matrices from layer S1 of the well-known model of the early visual cortex, HMAX [47], and measures of dissimilarity derived from the EEG responses at every time point [30]. Frequency decoding at 15 Hz can thus distinguish between those with healthy and those with abnormal early visual cortex functioning, such as JW.

Temporal decoding can also distinguish between other types of abnormal functioning, by highlighting when the EEG signal becomes unreliable: SM showed more reliable decoding early in the time course, with a peak decoding accuracy around 100 ms which is consistent with the responses from the healthy controls. JW, on the other hand, produced weak decoding early in the time course, but did produce reliable sustained decoding much later, after 400 ms. Both of these results are consistent with where the damage to the visual system is located. In JW, the damage to the early visual cortex bilaterally is consistent with poor early visual processing. In SM, the damage is later in the visual pathway in the temporal lobe, and so preserved early visual processing is consistent with this prognosis. While there are other methods than can be used to explore the temporal dynamics of the visual system, such as modulating presentation times [48], EEG is more sensitive to precisely when in the visual stream the EEG signal becomes un/reliable.

In addition to distinguishing between SM and JW, the temporal decoding offers some insight into how simple stimuli are encoded even when the early visual cortex is damaged. It is somewhat surprising that, in JW, decoding does eventually exceed chance. If signals being processed in the early visual cortex are degraded, then it is not obvious how these signals become stable with more time. There are several possible explanations for this. The first is that there is some top-down involvement that helps to regulate the incoming signal. JW has shown that he can learn to classify objects into different categories (Gabor patches with different spatial frequencies) visually and he can maintain this learning long term [31], demonstrating that while the visual signal is significantly degraded, it can still be useful for some complex visual processing. One of the explanations for the improved learning is that top-down mechanisms are able to generalize across trials, filling in the gaps of missing information and helping to restructure the incoming sensory information into a representation that is useable (for example, [49]). For JW, the damage is restricted to lower visual areas, while anterior visual areas are preserved, making this explanation plausible. The same may be true in the current study. The checkerboard alternates in contrast 15 times a second, and so by the time the decoding becomes stable in JW, he has already been exposed to five iterations of the checkerboard. The stimulus itself is relatively simple, and the only distinguishing features between the types of checkerboard is the spatial frequency. Therefore, five iterations may be sufficient to begin to stabilize the incoming information with the help of top-down mechanisms, potentially via stronger dorsal involvement which is relatively spared in JW compared to the ventral stream. Of course, this more stable extended signal is not obviously functional with regard to compensating for the agnosia as JW continues to evince a profound perceptual impairment even for simple visual inputs. It is also interesting to note that for JW decoding accuracy remained above chance after 400 ms, suggesting that the visual system was still responding to the stimulus. The controls, on the other hand, showed periodic accurate decoding, but little sustained activity. This could suggest that compensatory mechanisms take longer to encode stimuli, and could be a characteristic of impaired early visual functioning.

The second possible explanation for the recovered EEG decoding is that while early visual cortex is impaired, some partial signal is able to be encoded but may take many iterations to become stable, perhaps with the help of secondary visual areas. Rosenthal and Behrmann [31] also suggest a bottom-up mechanism for learning from the degraded visual signal, that focuses on retraining of receptive fields that may have shrunk due to the ischemic damage. The result of the shrinking receptive fields is a loss of global processing, that can be retrained with help from secondary visual areas. This potential mechanism cannot explain the results from the current study, as the training would take much longer than the duration of the stimulus. However, it is possible that the initial involvement of secondary areas that helps start the retraining of the visual system may be causing the delayed recovery of the EEG signal. With EEG alone, we are unable to tease apart the top-down versus bottom-up alternatives. Due to a prosthetic metal sternum in JW, we cannot obtain MRI data in order to establish whether more anterior visual areas are more involved in JW’s visual processing.

SM, on the other hand, shows intact early sensory processing but an inability to comprehend global object structure [34] or to learn object categories (multi-part Fribbles in this case) even with training [50]. Together, this demonstrates that while early visual processing is intact, higher visual areas are impacted. This matches the decoding results from his EEG responses: the classifier was able to decode SM’s EEG early in the time course, showing the same accuracy peak at 100 ms as healthy individuals, but decoding returned to chance later. It is important to note that SM’s decoding, although lower than most of the controls, was still within the control range. It is likely that due to the use of simple visual stimuli and high frequency presentation rates in this study, early visual processing dominated the signal. Future work could investigate temporal and frequency-domain decoding of high-level visual objects in SM. 

These findings complement the behavioral profiles of JW and SM: low-level visual processing is impaired in JW even though higher-level processing is less impaired, in contrast with SM who shows intact low-level visual processing, but deficits in more complex visual processing. However, the recovery of some of the EEG signal in JW may recommend time course analyses: the recovered visual signal later in visual processing in a damaged system may be difficult to obtain behaviorally.

### 4.2. Utility of EEG in the Study of Agnosia

The presence of two distinct patterns of EEG decoding accuracy in SM and JW corresponds to the site of the lesion in the visual system and complements previous behavioral findings of the extent to which early versus late visual signals are usable for processing visual information. While other imaging modalities such as MRI and MEG may be more helpful, faster, and cheaper for ascertaining where in the brain the damage is, these techniques may not be viable for individuals with metal in their bodies, such as JW. The use of EEG and a paradigm that allows assessment of visual responses in both time and frequency domains provides converging evidence of visual system impairments in individuals with agnosia.

Agnosia has been standardly subdivided into the two main subtypes, apperceptive agnosia and associative agnosia, each assumed to be the consequence of damage to either one of the two stages of processing of visual input. Although this binary distinction has long served as the framework for understanding agnosia, a number of challenges to this nomenclature have been offered with the specific claim that visual form processing is subserved by a series of computations rather than simply by two distinct stages. Consistent with this idea of more graded processing of inputs, one might then predict that there might be cases of visual agnosia whose symptoms do not match either of the two established subtypes and, indeed, there have been reports in which the patients’ deficits do not fall cleanly into either the associative or apperceptive stages. As an example, cases of agnosia have been described in whom lower-order perception is normal (patients can copy and match visual displays normally). These patients, however, are unable to integrate parts of visual inputs into a coherent holistic representation and this disorder has come to be labeled ‘integrative agnosia’ [10,51,52]. HJA, a well-studied integrative agnosic individual, illustrates the profile of integrative agnosia in his response to a photograph of a pepper shaker (or pepper pot): “a stand containing three separate pans; the top has a design on its lid; the second pan has a slightly smaller diameter than the top pan; the bottom pan has a wider diameter than the second pan and is longer in length” [53].

The temporal precision of EEG, the effective use of decoding methods, and the demonstration in this paper of the feasibility of differentiating between the electrophysiology of the two agnosic patients recommends the use of decoding EEG signals to help uncover the multiple different subtypes of agnosia, and how impaired visual processing at different levels of the visual system may differ between the subtypes. Particularly when compared with non-agnosic occipital lesion patient, multivariate EEG analyses may provide the differential temporal signatures associated with damage to different components of the cortical visual system. A future study contrasting decoding to low-level inputs like checkerboards and more complex patterns or objects might further differentiate between patients and their visual abilities.

### 4.3. Summary

Here, we have demonstrated that EEG decoding, by using the time course of the EEG signal, can be employed to infer where along the visual pathway the damage is located in individuals with agnosia. A significant advantage of EEG decoding methods is that they produce reliable results from single individuals to compare against a normal distribution of responses, which highlights the potential of using EEG and decoding methods in the neuropsychological cases, and potentially even in the medical setting, to ascertain the extent of brain damage.

In addition to the paradigm we have adopted, methods to use EEG to uncover spatial information have already shown some success. Dmochowski et al. [54] used Reliable Components Analysis on SSVEP signals to generate topographical maps that locate the spatial origin of the signal. This analysis utilized the high signal-to-noise ratio from the SSVEPs to maximize the trial-to-trial differences in signal. Similarly, using Representational Similarity Analysis, Kaneshiro et al. [55] found that the temporal information from single trial presentations of objects can help reconstruct spatial maps that are supported by fMRI results. Also, recent innovations by Murray and colleagues [56] have demonstrated that EEG can be recorded from patients at their bedside and that through their single-trial classification methods, heterogeneity among individuals as well trial-to-trial variability within subjects and patients can be evaluated [57,58]. Together, our findings as well as these demonstrations support the use of multivariate analyses on EEG signal to recover spatial information.

In summary, EEG can be useful when understanding where in the visual pathway the damage may be by focusing on when the EEG signal is perturbed or degraded. Focusing on the time course of visual processing also offers some insight into how visual information may be recovered when the damage is early in the visual stream (as in the case of JW). Together, the use of precise temporally-recorded data and cutting-edge analytics reveals the utility of this approach for investigating visual disorders such as agnosia, and for elucidating the temporal signature associated with visual perception.

## Supplementary Materials

The following are available online at http://www.mdpi.com/2411-5150/2/4/44/s1, Figure S1: Event-related potentials, Figure S2: Decoding results by visual field, Figure S3: Refiltering.
