# Supplementary material for: Differentiation of Types of Visual Agnosia Using EEG
Source: Vision (Basel). 2018 Dec 18;2(4):44. doi: 10.3390/vision2040044 (PMC6836011; doi:10.3390/vision2040044)
Supplement: Supplementary file 1 [file vision-02-00044-s001.pdf]

Supplementary material

## S1. Event-related potentials

Event-related potentials (ERPs) were calculated for stimuli for each visual field (mean of low, medium and high spatial frequency stimuli). For each patient and control participant, the median signal was taken from the left and right clusters of electrodes separately. Figure S1.1 shows ERPs for JW, SM and mean of the controls, shown separately for the different visual field stimuli and left and right electrode clusters.

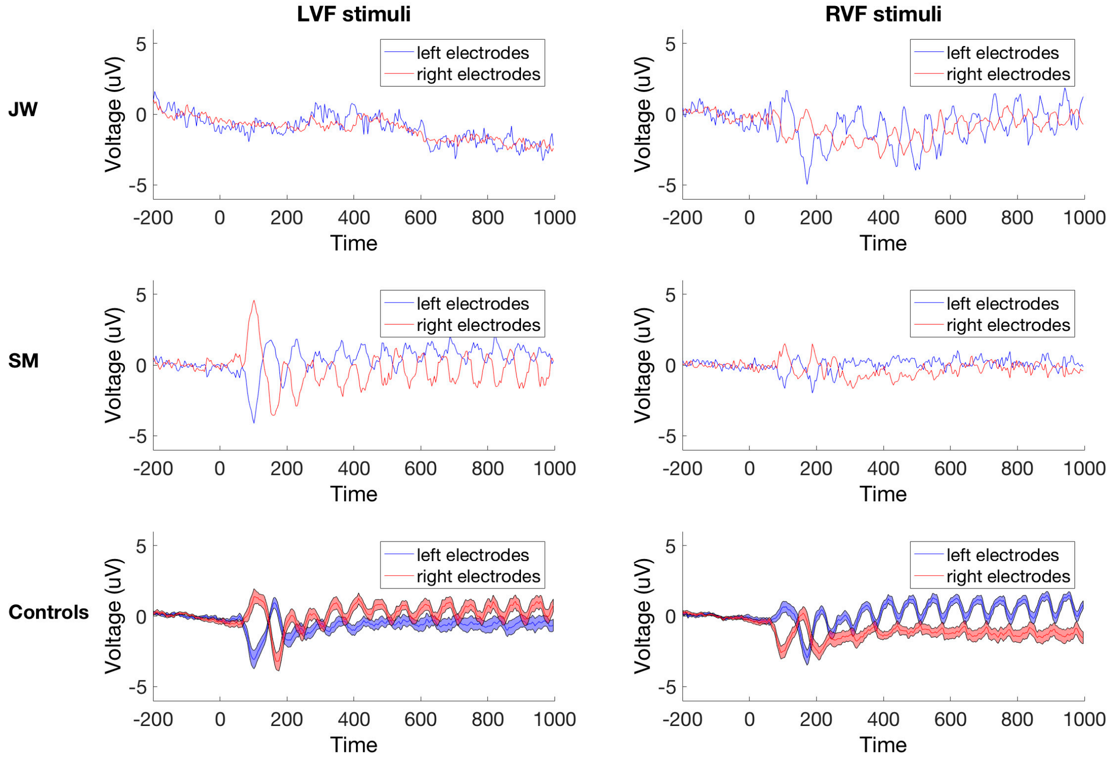


Figure S1.1 Event-related potentials for JW (top), SM (middle) and control group (bottom), plotted separately for mean left visual field stimuli (LVF; left) and right visual field stimuli (RVF; right).

For controls, electrodes over the contralateral hemisphere showed the largest periodic signal relative to the ipsilateral hemisphere, as expected. For both patients, there were hemispheric effects such that stimuli in one visual field resulted in larger signal. For JW, the signal was larger for the RVF stimuli than LVF stimuli, potentially due to his scotoma in the upper left visual field. For SM, the signal was larger for LVF than RVF stimuli, which was unexpected due to his right hemispheric lesion. It is clear, however, that the signal was stronger for the LVF stimuli for both the left and right electrodes, indicating that the differences were not due to electrode signal-to-noise ratio on one side.

S2. Decoding results by visual field

To assess hemispheric differences in the frequency domain decoding results, we calculated the mean pairwise decoding for stimuli within each visual field and across visual fields. The left visual field decoding, for example, consists of the mean of the LVF-low versus LVF-medium, LVF-low versus LVF-high, and LVF-medium versus LVF-high spatial frequency decoding results. The across visual field decoding consists of the mean of the LVF-low versus RVF-low, LVF-medium and RVF-medium, and LVF-high and RVF-high matched decoding pairs. The results are shown in Figure S2.1. JW had better decoding for RVF stimuli compared with LVF stimuli, in accordance with his ERPs and a potential result of his upper LVF scotoma. For SM, decoding was larger for LVF than RVF stimuli, much like the ERPs. For controls, decoding was similar for LVF and RVF stimuli. In both patients and controls, the across visual field results showed above-chance decoding, but it was clear that the mean pair decoding results presented in the paper (Figure 4) were not exclusively driven by across visual field effects.

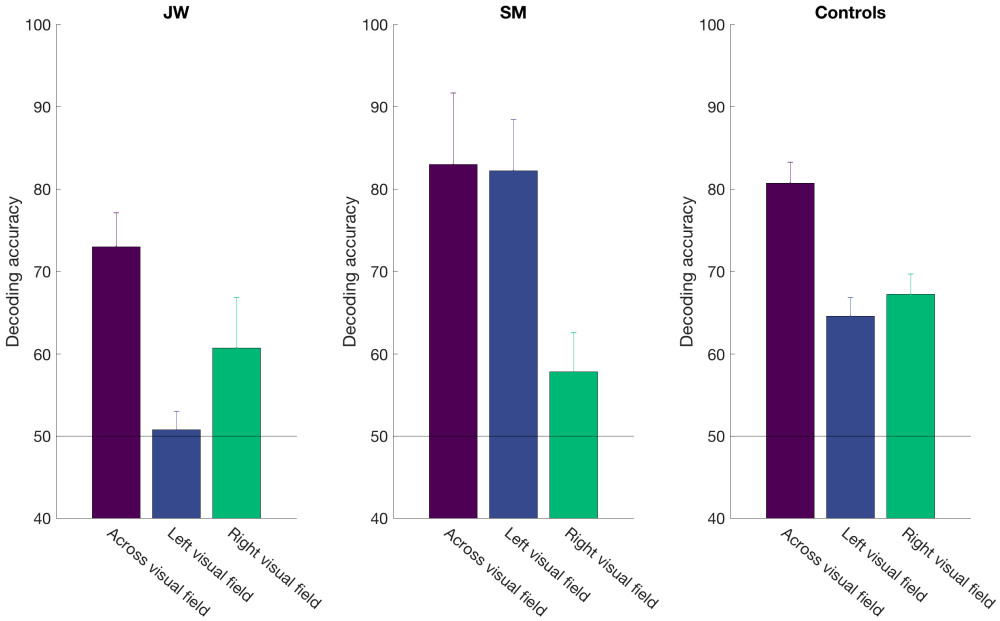


Figure S2.1 Frequency domain decoding for patients JW and SM, separated by decoding within the left and right visual fields, and across visual field. Error bars for JW and SM signify standard error of the mean across the three decoding pairs within each comparison, and error bars for controls signify standard error of the mean across the group (*N* = 16).

S3. Refiltering

To eliminate the influence of any non-brain artefacts to our time course decoding results, we re-ran the analyses using a more stringent filtering strategy. Continuous data were preprocessed as before, but were filtered using 10Hz high-pass and 20Hz low-pass filters. These cutoffs were chosen to be much more stringent around the frequency of interest (15Hz) but also to include other frequencies that might be informative for discriminating stimuli, such as alpha oscillations. Importantly, however, this filtering strategy is likely to remove low frequency artefacts such as blinks and eye movements, and high frequency muscular artefacts. The new analyses revealed similar results to the original analyses.

For the frequency domain decoding, both JW and SM exhibited significant decoding (Figure S3.1). JW had significantly poorer decoding than SM, however. These results were very similar to the analyses presented in the paper.

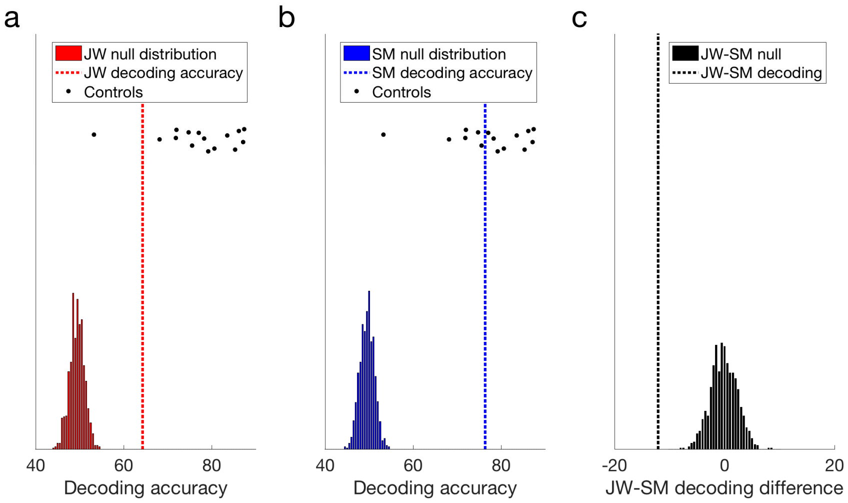


Figure S3.1 Frequency domain decoding results for JW, SM and the control group, after using more stringent filtering.

For the time course decoding, JW had much poorer decoding and was delayed relative to SM and controls (Figure S3.2). SM's classification performance was very similar to controls. Notably, JW's accuracy did not improve over the course of the trial as it did in the original analysis, although it remained above chance. It is important to note here, however, that the more stringent filtering strategy would have also removed neural signals that were not periodic within trials. This means that, for instance, neural responses to the first checkerboard onset in the trial, which are larger than subsequent checkerboard onsets, are dampened using this filtering strategy. Thus, if JW does require a large evidence accumulation window (for instance) to recognize images, resulting in higher decoding over time in the original analysis, it is likely that these filters will also remove these slow effects.

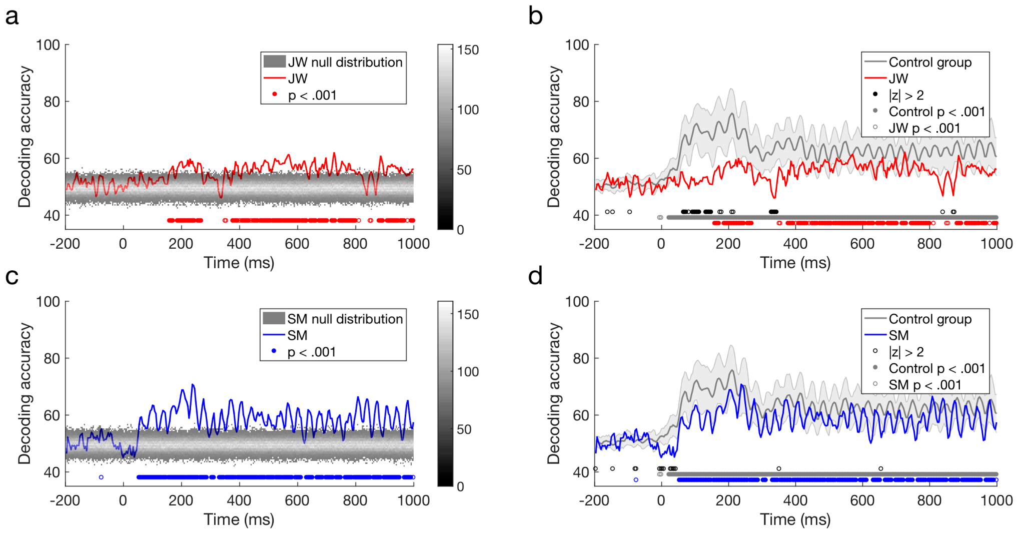


Figure S3.2. Time course decoding results for two agnosia patients. a) JW's decoding relative to shuffled label permutations. b) JW's decoding relative to control group. c) SM's decoding relative to shuffled label permutations. d) SM's decoding relative to the control group. Circles under plots represent significant differences between obtained accuracy and permutations or controls.

The difference in decoding between JW and SM is shown in Figure S3.3. SM clearly outperformed JW from approximately 100ms, and periodically for the rest of the epoch.

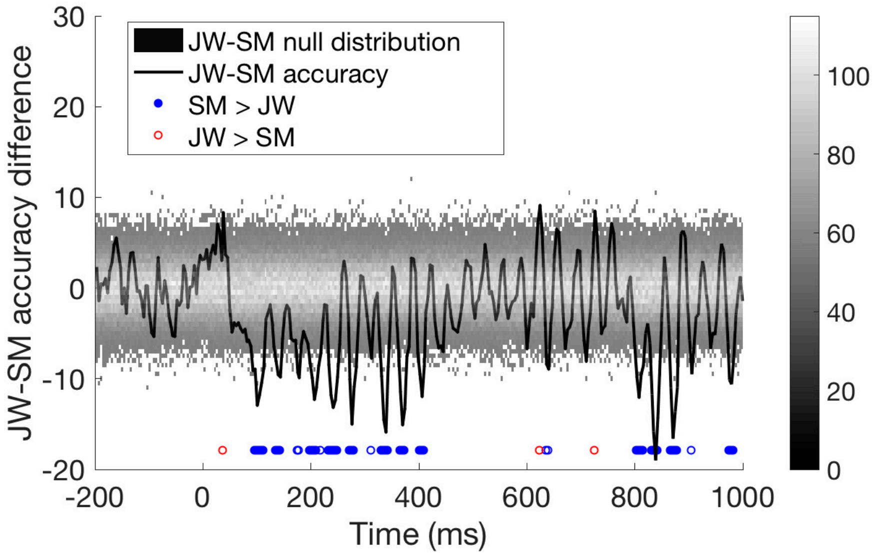


Figure S3.3. Difference in decoding between JW and SM relative to the difference in the permutations.

Finally, we assess the time course decoding at two separate timepoints (100ms and 600ms; JW had significant decoding at 600ms but not 100ms, whereas SM showed significant decoding at both times. Furthermore, SM outperformed JW at 100ms but not 600ms.

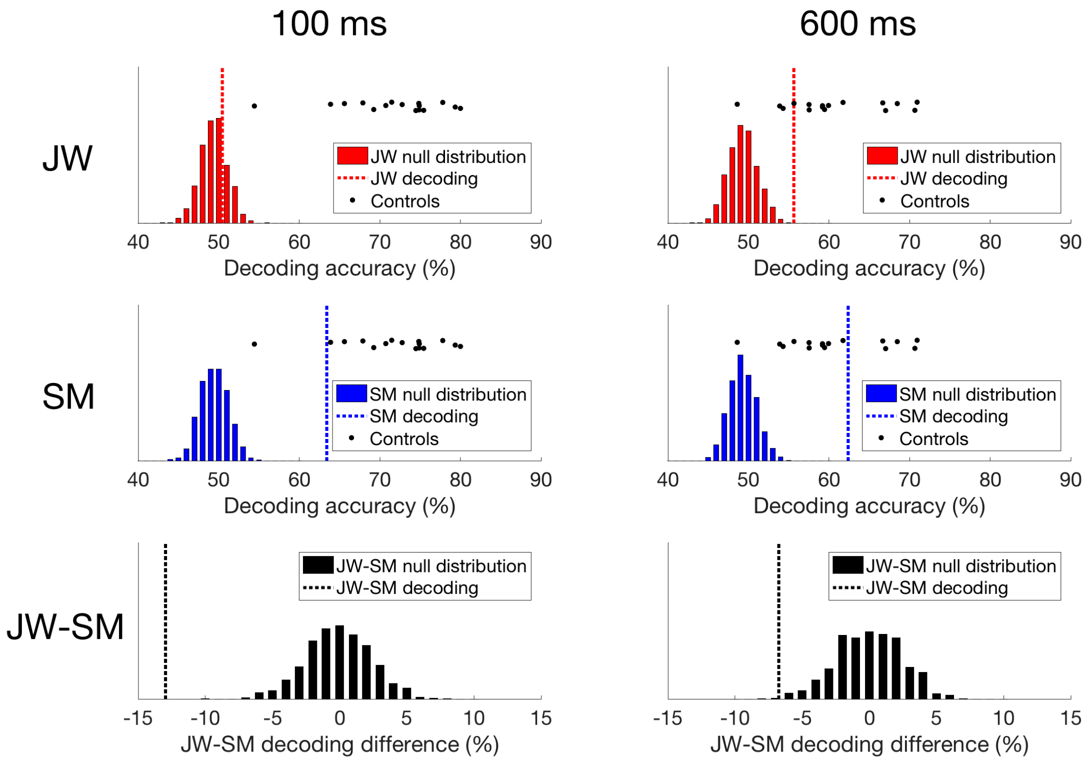


Figure S3.4. Time course decoding results at 100ms and 600ms after image onset for JW, SM and the difference between JW and SM.
